# Implications and Mechanisms of Antiviral Effects of Lactic Acid Bacteria: A Systematic Review

**DOI:** 10.1155/2023/9298363

**Published:** 2023-12-12

**Authors:** Fargol Farahmandi, Parynaz Parhizgar, Parya Mozafari Komesh Tape, Fahimeh Bizhannia, Fateme Sadat Rohani, Marzieh Bizhanzadeh, Zeinab Sadat Mostafavi Alhosseini, Maede Hosseinzade, Yeganeh Farsi, Mohammad Javad Nasiri

**Affiliations:** ^1^Student Research Committee, School of Medicine, Shahid Beheshti University of Medical Sciences, Tehran, Iran; ^2^Department of Microbiology, School of Medicine, Shahid Beheshti University of Medical Sciences, Tehran, Iran

## Abstract

**Background:**

Lactic acid bacteria (LAB) are among the most important strains of probiotics. Some are normal flora of human mucous membranes in the gastrointestinal system, skin, urinary tract, and genitalia. There is evidence suggesting that LAB has an antiviral effect on viral infections. However, these studies are still controversial; a systematic review was conducted to evaluate the antiviral effects of LAB on viral infections.

**Methods:**

The systematic search was conducted until the end of December 17, 2022, using international databases such as Scopus, Web of Science, and Medline (via PubMed). The keywords of our search were lactic acid bacteria, Lactobacillales, Lactobacillus (as well as its species), probiotics, antiviral, inhibitory effect, and virus.

**Results:**

Of 15.408 potentially relevant articles obtained, 45 eligible in-vivo human studies were selected for inclusion in the study from databases, registers, and citation searching. We conducted a systematic review of the antiviral effects of the LAB based on the included articles. The most commonly investigated lactobacillus specie were *Lactobacillus rhamnosus* GG and *Lactobacillus casei*.

**Conclusion:**

Our study indicates that 40 of the selected 45 of the included articles support the positive effect of LAB on viral infections, although some studies showed no significant positive effect of LABs on some viral infections.

## 1. Introduction

Viral infections have long been of great concerns, and emerging life-threatening viral infections during recent years have highlighted their importance [1]. Notable costs of treatment, considerable morbidity and mortality, and possible resistance to chemical drugs have led healthcare providers to seek alternative or adjuvant treatments to improve the cost-effectiveness of treatments and make them more available. Probiotics are among the most popular adjuvant treatments having proven effectiveness in a wide variety of diseases [2, 3].

Among probiotics, lactic acid bacteria (LAB) are among the well-studied bacteria, especially in recent years [4–6]. They are Gram-positive, non-spore-forming bacteria [7]. Some LABs exist as the normal flora of human and animal mucous membranes and colonize the gastrointestinal system (GI), skin, urinary tract, and genitalia [4]. LABs have several beneficial roles. In the GI tract, they reduce lactose intolerance, and they have antidiarrheal, anti-inflammatory, and antineoplastic activity [8]. As well, they have protective roles against peptic ulcers by eradicating *H. pylori* infection [9]. Modulation of immune responses and minimizing the allergic responses are their other beneficial effects [10, 11].

Growing evidence supports the antiviral, antibacterial, and antifungal effects of LABs [4, 5, 12]. Several mechanisms of antiviral activity have been proposed, both generally and specifically, according to certain viruses, but there is no conclusive research to establish the antiviral effects of LABs so far. The present study aims to systematically review the current literature on the antiviral effects of LABs and provide a comprehensive picture of it.

## 2. Materials and Methods

### 2.1. Search Strategy

This study was designed and performed to investigate the antiviral effects of LABs based on the Preferred Reporting Items for Systematic Reviews and Meta-Analyzes (PRISMA) checklist [13].

The systematic search was conducted until the end of December 17, 2022, in Scopus, Web of Science, and Medline (via PubMed). The searched keywords were a combination of the following:

Lactic acid bacteria, Lactobacillales, *Lactobacillus* (as well as its species such as: *Lactobacillus rhamnosus*, *Lactobacillus reuteri*, *Lactobacillus leichmannii*, *Lactobacillus helveticus*, *Lactobacillus fermentum*, *Lactobacillus brevis*, *Lactobacillus delbrueckii*, *Lactobacillus plantarum*, *Lactobacillus casei*, *Lactobacillus acidophilus*, *Lactobacillus sakei*, *Lactobacillus pentosus*, *Lactobacillus crispatus*, *Lactobacillus johnsonii*, *Lactobacillus gasseri*, *Lactobacillus salivariu*s, and *Lactobacillus paracasei*), probiotics, antiviral, inhibitory effect, virus.

### 2.2. Inclusion and Exclusion Criteria

All in-vivo original articles investigating the antiviral effects of LABs were considered eligible. Only studies written in English were included. Animal studies, in vitro studies, case reports, reviews, editorials, commentary, correspondence, and conference articles were excluded.

### 2.3. Study Selection

All search and screening steps were performed separately by two independent reviewers. The quality of articles included for data extraction was assessed by the Cochrane tool for experimental research and the Newcastle-Ottawa Scale (NOS) for observational studies [14, 15]. A third reviewer's opinion was obtained in case of disagreement (shown in Tables 1 and 2).

The NOS scale assesses the likelihood of bias in prospective studies using the following three domains: participant selection, comparability, and results. A research may receive up to one point for each numbered item in the selection, two points for comparability, and up to two points for outcome categories. For poor, moderate, and excellent study quality, corresponding scores of 0–3, 4–6, and 7–9 were given.

The Cochrane tool is based on the following: the use of random sequence generation, concealment of condition allocation, blinding of participants and staff, blinding of outcome assessors, completeness of outcome data, and other biases. Each study was given a risk of bias rating: low when there was no worry, uncertain when there was no information, and high when there was concern.

### 2.4. Data Extraction

Two authors independently extracted the following variables from studies included in this review: the first author's name, year of publication, type of study, the country of study execution, number of cases and controls, type of bacteria, type of virus, and antiviral effects of lactic acid bacteria on the virus (es).

## 3. Results

**Figure 1:**
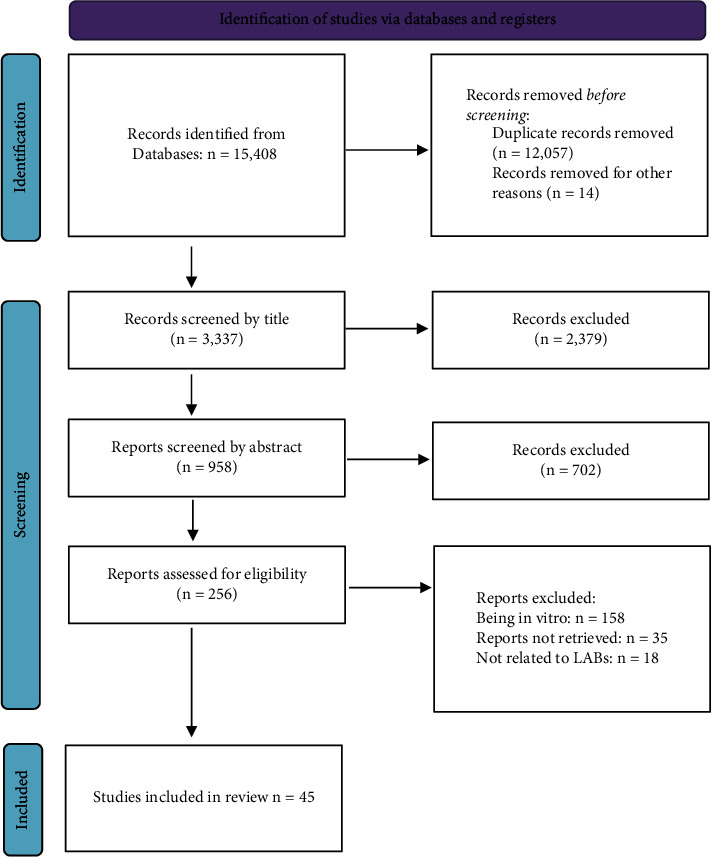
The study selection flowchart.

The study selection process is shown in Figure 1. According to the flowchart, 15,408 articles were obtained by the primary search. Due to duplication, 12,057 articles were excluded and the 3,337 remaining articles were screened.

In the next step, the title and abstract of the articles were screened in terms of being in vitro or in vivo, as well as the type of research article. Studies that seemed to be in vitro studies, case reports, reviews, editorials, commentary, correspondence, and conference articles were excluded (*n* = 3081). After screening the title and abstract, 256 articles remained for full-text screening. Ultimately, after exclusion of irrelevant and unavailable studies, 45 eligible articles were selected for full-text data extraction.

Of 45 included articles, 30 were randomized studies, 6 were cohorts, 7 were clinical trials, 1 case-control, and 1 placebo controlled crossover study.

Six of the included articles were conducted in Finland, six in Italy, four in Japan, three in the USA, three in Tanzania, three in China, two in Canada, two in Korea, two in Bolivia, two in UK, two in Belgium, one in each of the Vietnam, Egypt, Iran, Taiwan, Bangladesh, South Africa, India, Indonesia, Mexico, and Agra.  Table 3 provides more details about the final included articles.

### 3.1. LABs and Antiviral Effects

*L. rhamnosus* GG and *L. casei* were the most LAB probiotics investigated in the literature. While most of the articles confirmed the antiviral effects of LABs, some evidence did not support this idea.  Table 4 summarizes the antiviral effects of LABs, including the present application, their effects on viral load/shedding, clinical outcomes, and laboratory modifications are attributable to LABS (Table 4). The antiviral mechanism proposed in the studies was summarized into three major categories: (1) direct effect of LAB on viruses (the most common mechanism), (2) production of antiviral compounds, and (3) stimulation of the immune system against viruses. These mechanisms are discussed in more detail subsequently.

Another notable aspect of our study is the antiviral effects of LABs in immunocompromised patients. While most of the postulated mechanisms have been about immunocompetent individuals, studies in immunocompromised patients, such as HIV+ patients, dedicate activation of immune cells such as CD4+ cells, amelioration of inflammation, and decrease in translocation markers. The attributable mechanisms would be explained subsequently.

## 4. Discussion

**Figure 2:**
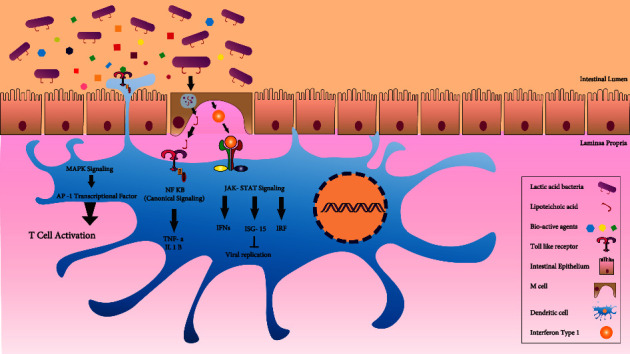
A summary of the mechanisms of the antiviral effects of LABs. The first process involves interactions between LABs and viruses that trap or adhere viruses to the surface of the host cell, preventing them from adhering to cells. The generation of bioactive antiviral substances such as H2O2, lactic acid, bacteriocins, exopolysaccharides, etc., is the second mechanism. The third mechanism involves interferon-associated mechanisms, which include (a) activation of dendritic cells, which results in T-cell activation, production of cytokines, and inhibition of viral replication; (b) activation of the JAK-STAT signaling cascade, which results in transcription of genes with antiviral and immunomodulatory effects, as well as binding of ISG15 to viral proteins, which causes them to be destroyed; and (c) binding of bioactive substances to the toll-like receptor.

As we have stated so far, five of the selected 45 publications revealed no discernible impact on the viruses. These five articles were administered L. rhamnosus; two had *L. helveticus*, and one had *B. animalis* spp., Lactis in their administered probiotics in addition. The major explanation of this discrepancy could be the limited research population, the pathogen change, the comparison to vaccination, the varied study techniques from earlier studies, and the various months of patient enrollment in the study. According to the current evidence, central mechanisms of LAB antiviral effects can be categorized into three major groups: (1) the direct interactions between LABs and viruses, (2) the production of bioactive antiviral agents, and (3) the induction of interferon-associated mechanisms. We have summarized these mechanisms in Figure 2 and discuss each mechanism in more detail.

1. The direct interactions between LABs and viruses
2. Direct interactions between the LABs and viruses are the primary mechanism of virus inactivation. The direct antiviral effect of LAB is applied via both adsorption and trapping, which are strain-dependent mechanisms and can inhibit viruses in a nonspecific and perhaps specific manner [5, 61]. Botić et al. showed that *L. paracasei*, *L. rhamnosus*, *L. plantarum*, and *L. reuteri* could trap vesicular stomatitis virus (VSV) [61]. Also, *L. gasseri* has direct antiviral ability against simplex type 2 (HSV-2) [62]. Some *Lactobacillus* strains can trap HIV virions by binding their glycoprotein gp120 to the mannose sugar-rich “dome” at the end of the HIV attachment proteins. These results suggest the existence of identical mechanisms in inhibiting viruses by LAB strains [63]. Notably, attaching LABs to the cell surface leads to blocking the binding of viruses to their receptors on the cell surface, preventing viruses from entering the cell in the early stages of infection [64, 65].
3. The production of bioactive antiviral agents
4. Hydrogen peroxide (H_2_O_2_) and lactic acid produced by *Lactobacillus* spp. are two critical antimicrobial products of LAB. Both have antiviral effects against HIV-1 and HSV-2 via acidifying the pH of their microenvironment [66–69]. Bacteriocins are other groups of LAB products that play essential roles in destroying virus-infected cells via diverse mechanisms, such as creating holes in target cell membranes, lowering intracellular pH, and blocking enzymatic activities [70]. It is shown that *L. delbruecki* developed a noncytotoxic bacteriocin that was found to be virucidal against the influenza virus [71]. Exopolysaccharides (EPS) derived from the genus *Leuconostoc* spp. have shown antiviral activities against HSV-1 [72]. The EPS applies strong virucidal action and reduces the viral adsorption, penetration, and production of virus particles by 97–99%; EPSs 2t and 19s decrease viral progeny infectivity by blocking viral adsorption to cells [73]. EPS 26a is another family member with proven inhibitory effects on Human Adenovirus Type 5 (HAdV-5) reproduction [74]. EPS 26a has strong anti-HAdV-5 action by developing noninfectious viral offspring [74]. Other bacterial components, such as a non-protein-composed wall component, inhibit viral reproduction [75]. In cell cultures, a nonprotein cell wall component isolated from a vaginal strain of *L. brevis* significantly inhibited HSV-2 replication [75].
5. Induction of interferon-associated mechanisms
6. LABs stimulate immune system components via lipopolysaccharide (LPS), lipoproteins, lipopeptides, and unmethylated CpG motifs mainly through toll-like receptors [76, 77].

Moreover, M cells ingest LABs, producing interferon type I (type I IFN), which activates dendritic cells [78]. Activated dendritic cells trigger multiple critical intracellular signaling pathways, resulting in T-cell activation, viral replication inhibition, and production of immunomodulatory cytokines [79].

Production and secretion of IFNs provide a high level of antiviral protection [80]. The Janus kinase-Sign Transducer and Activator of Transcription (JAK-STAT) signaling cascade is activated by type I and type III IFNs, which phosphorylate STAT1 and STAT2 [81]. Phosphorylated STATs and the IFN Regulatory Factor-9 (IRF9) form the IFN-Stimulated Gene Factor 3 (ISGF3) complex [80]. The ISGF3 complex enters the cell nucleus and enhances the transcription of genes that have IFN-Stimulated Response Element (ISRE) in their promoters [80]. These genes might have antiviral or modulatory effects on the inflammation and cytokine production pathways [80]. Type II IFNs also cause STAT1 homodimerization, which favors ISG promoters with gamma-activated sequence (GAS) containing promoters [80]. All result in the induction and activation of several antiviral agents, including the protein kinase RNA-activated (PKR), ribonuclease 2-5A pathway, and numerous apoptotic pathways which inhibit the virus binding to cells, viral particle penetration into cells, and the release of the nucleocapsid from an envelope [82]. Disruption of transcription and translation processes of the structural viral proteins prevents virion formation or budding of viruses [83].

Another key mediator of IFN antiviral effects is interferon-stimulated gene 15 (ISG15), a ubiquitin-like protein that plays a significant role in counteracting viruses by conjugating to the viral and cellular proteins and marking them for destruction [84]. Three enzymes mediate the ISG15 conjugation with the target protein; E1 activating enzyme (Ube1L), E2 conjugating enzyme (UbcH8), and E3 ligase enzyme (either HERC5, or EFP, or TRIM25). These enzymes establish a covalent bond between the C-terminal glycine of the mature form of ISG15 and the target protein lysine [84–86]. Conjugation of ISG15 with viral proteins leads to impaired protein function, promotes protein degradation, and prevents oligomerization of viral proteins [87]. IFNs increase the poly-SUMOylation and ISGylation of both viral and cellular proteins [88]. As well, the ISG15 can modify and inhibit the viral mRNA translation by strengthening the attachment of translation suppressor proteins such as eIF4E homologous protein (4EHP) to the viral mRNA cap [89]. ISG15 is also secreted from various cells [90]. The extracellular ISG15 can also act as an antiviral cytokine by producing and secreting antiviral factors such as type III IFNs, nitric oxide (NO), and reactive oxygen species (ROS), which induce apoptosis in virus-infected cells [87, 91, 92]. Binding ISG15 to the lymphocyte function-associated antigen 1 (LFA-1), located on immune cells, results in cell proliferation, maturation, and production of IFN-*γ* and IL-10 [93–97]. Bioactive agents and LAB particles are essential ligands for toll-like receptors (TLRs) [98]. TLRs' activation results in T-cell activation and cytokine production via mitogen-activated protein (MAP) kinases and NF-kB signaling pathways in dendritic cells [99]. MAP kinases directly phosphorylate the transcription factor AP-1, a key player in T-cell activation [100–102]. The phosphorylated AP-1 heterodimerizes in the nucleus and binds to the IL-2 promoter and enhance its expression which promotes the T-cell activity [102].

## 5. Conclusion

According to the current literature, LABs have considerable antiviral activities which affect viruses both directly and indirectly. Although our study provides an overview of the antiviral activities of the LABs as one of the most important human gut microbiota, it has some limitations: the applicability of using LABs as antiviral adjuvants in clinical practice has not been fully investigated, and so it is not justifiable by our study. As well, the potential adverse effects of the therapeutic use of LABs and their delivery system as a therapeutic agent are not investigated. We propose further studies to investigate these concerns and promote our knowledge about more efficient antiviral agents.

## Figures and Tables

**Table 1:** Quality assessment of the observational studies included in the meta-analysis (the NOS tool).

Author	Selection				Comparability		Outcome			
	Representativeness of exposed cohort	Selection of nonexposed cohort	Ascertainment of exposure	Demonstration that outcome of interest was not present at start of study	Adjust for the most important risk factors	Adjust for other risk factors	Assessment of outcome	Follow-up length	Loss to follow-up rate	Total quality score
Li et al. [16]	1	1	1	1	1	1	1	1	1	9
Salazar et al. [17]	1	1	1	1	1	0	1	1	1	8
Ishizaki et al. [18]	1	1	1	1	1	1	1	1	1	9
Nagata et al. [19]	1	1	1	1	1	1	1	1	1	9
Irvine et al. [20]	1	1	1	1	1	0	1	1	1	8
Irvine et al. [21]	1	1	1	1	1	0	1	1	1	8
Salminen et al. [22]	1	1	1	1	1	0	1	1	1	8
Wang et al. [23]	1	1	1	1	1	1	1	1	1	9

**Table 2:** Quality assessment of the experimental studies included in the meta-analysis (the Cochrane tool).

Author	Random sequence generation	Allocation concealment	Blinding of participants and personnel	Blinding of outcome assessment	Incomplete outcome data	Selective reporting
Freedman et al. [24]	Low risk	Low risk	Low risk	Low risk	Low risk	Low risk
Shin et al. [25]	High risk	Low risk	Low risk	Low risk	Low risk	Some concern
Ou et al. [26]	Low risk	Low risk	Low risk	Low risk	Low risk	Low risk
Allam et al. [27]	High risk	Low risk	Low risk	Low risk	Low risk	Some concern
Palma et al. [28]	Some concern	Some concern	High risk	Low risk	Low risk	Some concern
Mohseni et al. [29]	Low risk	Low risk	Low risk	High risk	Low risk	Low risk
Wang et al. [30]	Low risk	Low risk	Low risk	Low risk	Low risk	Low risk
D'Ettorre et al. [31]	High risk	Low risk	Some concern	High risk	Low risk	Some concern
Fujii et al. [32]	Low risk	Low risk	Low risk	Low risk	Low risk	Low risk
Gleeson et al. [33]	Low risk	Low risk	Low risk	Low risk	Low risk	Low risk
Tapiovaara et al. [34]	Low risk	Low risk	Low risk	Low risk	Low risk	Low risk
D'Ettorre et al. [35]	Some concern	Some concern	Low risk	Low risk	Low risk	Low risk
Sugimura et al. [36]	High risk	Low risk	Low risk	Low risk	Low risk	Low risk
Niekerk et al. [37]	Low risk	Low risk	Low risk	Low risk	Low risk	Some concern
Lee et al. [38]	Low risk	Low risk	Low risk	Low risk	Low risk	Some concern
Lehtoranta et al. [39]	Low risk	Low risk	Low risk	Low risk	Low risk	Low risk
Luoto et al. [40]	Low risk	Low risk	Low risk	Low risk	Low risk	Low risk
Gautam et al. [41]	High risk	Some concern	Low risk	Low risk	Low risk	Some concern
Santiago et al. [42]	Low risk	Low risk	Low risk	Low risk	Low risk	Some concern
Kumpu et al. [43]	Low risk	Low risk	Low risk	Low risk	Low risk	Low risk
Verhoeven et al. [44]	High risk	Some concern	Low risk	Low risk	Low risk	Low risk
Hummelen et al. [45]	Low risk	Low risk	Low risk	Low risk	Low risk	Low risk
Nagata et al. [19]	High risk	Some concern	Low risk	Low risk	Low risk	Some concern
Grandy et al. [46]	Low risk	Low risk	Low risk	Low risk	Low risk	Low risk
Teran et al. [47]	Low risk	Low risk	Low risk	Low risk	Low risk	Some concerns
Fang et al. [48]	Low risk	Low risk	Low risk	Low risk	Low risk	Some concerns
Dubey et al. [49]	Low risk	Low risk	Low risk	Low risk	Low risk	Low risk
Matsuzaki et al. [50]	High risk	Some concerns	Low risk	Low risk	Low risk	Some concerns
Sarker et al. [51]	Low risk	Low risk	Low risk	Low risk	Low risk	Low risk
Mastretta et al. [52]	Low risk	Low risk	Low risk	Low risk	Low risk	Low risk
rWolf et al. [53]	Low risk	Low risk	Low risk	Low risk	Low risk	Low risk
Heli Majamaa [54]	Low risk	Low risk	Low risk	Low risk	Low risk	Some concerns
De Boeck et al. [55]	High risk	Low risk	Low risk	Low risk	Low risk	Some concerns
Dellino et al. [56]	Low risk	Low risk	High risk	Low risk	Low risk	Low risk
Di Pierro et al. [57]	Low risk	Low risk	Some concerns	Some concerns	Low risk	Some concerns
Gutiérrez-Castrellón et al. [58]	Low risk	Low risk	Low risk	Low risk	Low risk	Low risk
Koesnoe et al. [59]	High risk	Low risk	Low risk	Low risk	Low risk	Low risk
Mullish et al. [60]	Low risk	Low risk	Low risk	Low risk	Low risk	Low risk

**Table 3:** A summary of the included articles' characteristics.

The first author	Year	Country	Type of study	Probiotics	Virus	Mean age (years)	Study population (LAB recipient vs. control)
De Boek et al. [55]	2022	Belgium	RCT	*L.casei AMBR2*, *L.plantarum WCFS1*, and *L.rhamnosus GG*	SARS-CoV2	42 ± 12 (verum) 43 ± 12 (placebo)	60 (33 : 27)
Dellino et al. [56]	2022	Mexico	RCT	*L.plantarum KABP022*, *KABP023*, and *KAPB033*	SARS-CoV2	37.0	300
Castrellón et al. [58]	2022	Italy	CT	*L.crispatus* M247	Papilloma virus	30–65 years old	160
Dl Pierro et al. [57]	2021	Italy	Preliminary, uncontrolled, open trial	*L. crispatus M247*	HPV	NG	35
Koesnoe et al. [59]	2021	Indonesia	RCT	*L.helveticus R0052*, *L.rhamnosus R0011*	Influenza	67 ± 5.6	554
Mullish et al. [60]	2021	UK	CT	*L.acidophilus*, *L.plantarum*	SARS-CoV2	More than 45	220
Wang et al. [23]	2021	China	Cohort	*L.bulgaricus*	SARS-CoV2	48.58	156 (98 : 58)
Li et al. [16]	2021	China	Cohort	*B. infantis*, *L. acidophilus*, *Dung enterococcus*, *B. cereus* + *B. longum*, *L. bulgaricus*, *S. thermophiles* + *E. faecium*, *B. subtilis*	SARS-CoV2	60.1	311 (123 : 188)
Freedman et al. [24]	2020	Canada	RCT	*L.rhamnosus*, *L. helveticus*	Adenovirus, norovirus, rotavirus	NC	816 (408 : 408)
Shin et al. [25]	2020	Republic of Korea	RCT	*L. plantarum*	Rotavirus	NC	50 (15 : 35)
Ou et al. [26]	2019	China	RCT	*L.rhamnosus GR-1*, *L. reuteri RC-14*	HR-HPV	44.8	121 (62 : 59)
Allam et al. [27]	2019	Egypt	CT	*L. acidophilus*, *Bifidobacterium* spp.	HCV	48	40 (20 : 20)
Palma et al. [28]	2018	Italy	RCT	*L.rhamnosus*	HPV	30.7	117 (60 : 57)
Salazar et al. [17]	2018	USA	Cohort	*Lactobacillus* spp.	RSV	21.8 (weeks)	118 (118 : 0)
Mohseni et al. [29]	2018	Iran	RCT	*L. brevis*	HSV-2	36.7	66 (33 : 33)
Wang et al. [30]	2018	Canada	RCT (pilot)	*L.rhamnosus GG*	Influenza A, influenza B, entero-rhino virus, RSV, metapneumovirus, Parainfluenza 1, 2, and 3	85.5	196 (100 : 96)
D'Ettorre et al. [31]	2017	Italy	CT (sub study of a pilot)	*L. plantarum DSM24730*, *S. thermophilus DSM24731*, *B. breve DSM24732*, *L. paracasei DSM24733*, *L. delbrueckii* subsp. *bulgaricus DSM24734*, *L. acidophilus DSM 24735*, *B. longum DSM24736*, *B. infantis DSM24737*	HIV	42 (med)	10 (10 : 0)
Ishizaki et al. [18]	2017	Vietnam	Cohort	*Lactobacillus casei Shirota* (*LcS*)	HIV	NC	80 (80 : 0)
Fujii et al. [32]	2017	Japan	RCT	*L. lactis JCM 5805*	Influenza A (H1N1, H3N2)	38.6	107 (54 : 53)
Gleeson et al. [33]	2016	UK	RCT	*L. casei Shirota* (LcS)	HSV, EBV, CMV	20.4	243 (126 : 117)
Tapiovaara et al. [34]	2016	Finland	RCT	*L.rhamnosus GG*	Rhinovirus	NC	59 (39 : 20)
D'Ettorre et al. [35]	2015	Italy	CT	S. salivarius ssp. thermophilus, *S. faecium*, *B. breve*, *B. infantis*, *B. longum*, *L. acidophilus*, *L. plantarum*, *L.casei, L. delbrueckii* ssp. *bulgaricus*	HIV	49.7	31 (20 : 11)
Sugimura et al. [36]	2015	Japan	RCT	*L. lactis JCM 5805*	Influenza A (H1N1)	45.2	213 (106 : 107)
Niekerk et al. [37]	2015	South Africa	RCT	*L.rhamnosus GG,* Bifidobacterium infantis	HIV	Neonates	184 (91 : 93)
Lee et al. [38]	2014	Republic of Korea	RCT	*B. longum*, *B.lactis*, *L. acidophilus*, *L.rhamnosus*, *L.plantarum*, *P. pentosaceus*	Rotavirus	1.9	29 (13 : 16)
Lehtoranta et al. [39]	2014	Finland	RCT	*L. rhamnosus GG*, *B. animalis* ssp. *lactis BB-12*	Picornaviruses, HRV, HEV	NC	192 (90 : 102)
Luoto et al. [40]	2014	Finland	RCT	*L.rhamnosus GG*	Rhinovirus, RSV, Adenovirus, coronavirus 229E/NL63 and OC43/HKU1, influenza A and B, rhinovirus, Parainfluenza virus type 1, 2, 3, RSV group A and B, human metapneumovirus, human bocavirus, human enterovirus	Neonates	45^1^ (21 : 24)
Gautam et al. [41]	2014	Agra	RCT	*L. sporogens*	HIV	NC	107
Santiago et al. [42]	2013	USA	RCT	*Lactobacillales*	HIV	33	13
Kumpu et al. [43]	2013	Finland	RCT (sub study)	*L.rhamnosus GG*	HRV, HEV, ADV, RSV, influenza A (H1N1, H3N2), influenza B, parainfluenza virus type 1, 2, 3, human bocavirus	3.7	194 (97 : 97)
Verhoeven et al. [44]	2013	Belgium	CT	*L.casei*	HPV	31.77	51 (24 : 27)
Hummelen et al. [45]	2011	Tanzania	RCT	*L.rhamnosus GR-1*	HIV	NC	112 (55 : 57)
Nagata et al. [19]	2011	Japan	Case- control	*L. casei Shirota* (*LcS*)	Norovirus	84	77 (39 : 38)
Irvine et al. [20]	2011	Tanzania	Cohort (comparative, retrospective)	*L.rhamnosus*	HIV	38.5	171 (85 : 86)
Grandy et al. [46]	2010	Bolivia	RCT	*L. acidophilus*, *L.rhamnosus*, *B. longum*, *S.* boulardii	Rotavirus	NC	64 (26 : 50)
Irvine et al. [21]	2010	Tanzania	Cohort (comparative, retrospective)	*L.rhamnosus GR-1*	HIV	NC	150 (68 : 82)
Teran et al. [47]	2009	Bolivia	RCT	*L. acidophilus*, *L.rhamnosus B. longum*, *S. boulardii*	Rotavirus	9.3	75 (25 : 50)
Fang et al. [48]	2009	Taiwan	RCT	*L.rhamnosus 35* (*Lcr35*)	Rotavirus	2.7 (med)	23 (17 : 6)
Dubey et al. [49]	2008	India	RCT	*L. acidophilus*, *L. paracasei*, *L. bulgaricus*, *L. plantarum*	Rotavirus	NM	224 (113 : 111)
Matsuzaki et al. [50]	2005	Japan	Uncontrolled trial	*L. casei Shirota* (*LcS*)	HTLV-1	NM	10 (10 : 0)
Sarker et al. [51]	2005	Bangladesh	RCT	*L. paracasei ST 11*	Rotavirus	10 months	230 (115 : 115)
Salminen et al. [22]	2004	Finland	Placebo-controlled, crossover study	*L.rhamnosus GG*	NM (just virus causing diarrhea)	44.5	17 (8 : 9)
Mastretta et al. [52]	2002	Italy	RCT	*Lactobacillus GG*	Rotavirus	NC	220 (114 : 106)
Wolf et al. [53]	1998	USA	RCT	*L. reuteri*	HIV	NM	39 (20 : 19)
Majamaa et al. [54]	1994	Finland	RCT	*L.casei ssp. rhamnosus*, *L. casei* ssp. casei strain GG	Rotavirus	1.5	49 (30 : 19)

RCT: randomized controlled trial, CT: clinical trial, HR-HPV: high risk human papilloma virus, HCV: hepatitis C virus, RSV: respiratory syncytial virus, HSV-2: herpes simplex virus-2, HIV: human immunodeficiency virus, EBV: epstein barr virus, CMV: cytomegalo virus, HRV: human rhinovirus, HEV: human enterovirus, ADV: adenovirus, HTLV-1: human T-lymphotropic virus type 1, NC: not calculable, NM: not mentioned.

**Table 4:** Summary of the applied clinical conditions, effects of LAB supplementation on viral load/shedding, clinical signs, and symptoms in comparison with the standard treatment strategies.

The first author	Clinical condition	Effect on viral load/shedding	Effect on signs and symptoms	Para clinical assessments	Conclusion
De Boek et al. [55]	The clinical potential of multispecies throat spray against SARS-CoV2	Decreased viral load and lower number of virus positive test results	Symptom improvement specially in intranasal administration of probiotics	—	Change in the severity of symptoms, faster recovery and reduction of absolute level of SARS-CoV-2 particles and microbiome change in nose/throat
Dellino et al. [56]	Women affected by HPV infections	—	Reduced infection signs	Higher percentage of clearance of PAP-smear abnormalities	Potential effect on resolving cervical abnormalities
Castrellón et al. [58]	Symptomatic SARS-CoV2-infected adults	Complete viral clearance, lower nasopharyngeal viral load	Complete symptomatic clearance	Higher serum titers of SARS-CoV2-binding IgG and IgM	Increasing complete viral and symptomatic remission, reducing symptom duration, viral load and lung infiltrates while increasing SARS-CoV2-specific IgM and IgG
Dl Pierro et al. [57]	HPV-positive women	Reduction in HPV positivity	Significant change in CST status	Increased HPV clearance	Change in CST status and, in parallel, increased HPV clearance
Koesnoe et al. [59]	Healthy elderly subjects aged 67 ± 5.6	—	—	A significant increase in postvaccination seroprotection in groups receiving vaccines with probiotics and without probiotics but not in people who did not get vaccination, the antibody titers peaked out one month postvaccination	No reduction in the relative risk of ILI events was observed in vaccinated individuals, while probiotic supplementation did not influence seroprotection and seroconversion
Mullish et al. [60]	Healthy, free-living, overweight and obese adults	—	Reducing URTI symptoms in overweight and obese people	—	Reducing URTI symptoms in overweight and obese people
Wang et al. [23]	Cases of SARS-CoV2	—	Shorter duration of diarrhea in the probiotics group	Higher PCT and CR, plasma albumin and lymphocyte counts and shorter time of negative nucleic acid test	Safe and effective and early application is recommended
Li et al. [16]	Severe SARS-CoV2	—	Lower rates of secondary infections	Increased IL-6 and ESR	Effective augmenter of the immunity during the SARS-CoV2
Freedman et al. [24]	Acute gastroenteritis in children	No difference	No difference	—	Not effective
Shin et al. [25]	Rotaviral enteritis in infants	Decreased viral proliferation and shedding in stool	Decreased diarrhea, improved Vesikari score	—	Effective
Ou et al. [26]	HR-HPV colonization	No significant difference in the clearance rate	—	Decreased abnormal/unsatisfactory pap smear results	Not effective in decreasing the clearance rate but effective in improvement of the pathology
Allam et al. [27]	Antiviral and immune system improvements of HCV patients	Significant decrease	Protection against the most 5 common bacteria infect the HCV patients	—	Effective anti-viral and antibacterial activity
Palma et al. [28]	Anti-HPV effects in women with dysbiosis and concomitant HPV-infections	Higher rates of HPV clearance	—	Improved HPV related anomalies in pap smear	Effective
Salazar et al. [17]	Acute respiratory infection in infants	—	Absence of wheezing at the age of 2 years	—	Reduced risk of childhood wheezing illnesses at age 2 years
Mohseni et al. [29]	Recurrent genital HSV-2 infections	Similar results in decreasing the viral shedding	The comparable effects in, resolution of episode, lesion healing time and percentage of pain	—	Suppression of the recurrent infection
Wang et al. [30]	Laboratory-confirmed respiratory viral infections	—	No difference between infection rate or the severity of illness	—	Further investigations are needed
D'Ettorre et al. [31]	Gut mucosal integrity in HIV-1 infected patients receiving ART	—	—	Reduced CD4+, CD8+ T‐cell, increased Th17 cells, improved integrity of the gut epithelial barrier, reduction of intramucosal lymphocyte infiltration and enterocyte apoptosis, improved mitochondrial morphology	Restoring the physical and immunological integrity of the mucosal intestinal barrier in ART‐treated HIV + patients
Ishizaki et al. [18]	Immunologic profile, intestinal bacterial translocation, paraclinical assessments in HIV + ART +, HIV + ART -, and HIV - children	Decreased viral load in HIV + ART- subgroup	Increased height and weight	Improved LFT, decreased Hb, increased PLT, increased Th2, Th17, and decreased CD8+ activity, without significant improvements in bacterial translocation	Safe and effective
Fujii et al. [32]	The influenza viral infection in healthy adults	—	Reduction in the severity of symptoms such as sore throat	Increased secretory IgA, phagocytic activity, antiviral gene expression	Enhanced immunity and immune related mechanisms
Gleeson et al. [33]	URI symptoms and Ab response in healthy adults	—	No change in the rate of infection	Decreased anti-CMV and anti- EBV antibody	Reduced plasma CMV and EBV antibody titers
Tapiovaara et al. [34]	HRV viral load in healthy individuals	No difference	—	—	No difference
D'Ettorre et al. [35]	Immune system activation and function in HIV-1 infected adults	—	—	Lower CD4+ count but higher immune system activation in HIV patients	Reduced mucosal and systemic inflammation
Sugimura et al. [36]	Pathogenesis and immune response to influenza virus in healthy adults	—	Amelioration in symptoms severity	Increased IFN-a, increased CD86	Protection against the pathogenesis of an influenza-like illness
Niekerk et al. [37]	The incidence of NEC in premature neonates born from HIV+ and HIV- mothers	—	Decreased NEC in probiotic recipients of both HIV+ and HIV- groups, reduced severity of disease in the HIV-exposed study group	—	Reduced the incidence of NEC in the premature very low birth weight infants
Lee et al. [38]	Rotaviral diarrhea in children	—	Decrease the duration of diarrhea and vomiting	No significant difference	Safe and effective
Lehtoranta et al. [39]	Nasopharyngeal colonization of respiratory viruses in children	Reduced the mucosal colonization of picornaviruses after 3 months	No significant reduction in symptomatic cases	—	Not protective against the incidence of infection
Luoto et al. [40]	Viral URI in preterm neonates	—	Lower incidence of URI, no difference in duration/severity of symptoms	—	Protective against viral URI incidence in preterm neonates
Gautam et al. [41]	Immunologic and clinical outcomes of probiotics in HIV-infected children	—	—	Increased CD4+	Increased CD4+ counts
Santiago et al. [42]	Targeting the alterations of gut microbiota before and after ART in HIV-1 infected patients	Negative correlation between proportion of *Lactobacillales* and viral load	—	HIV-infected individuals were associated with lower markers of microbial translocation and during ART, higher proportions of gut Lactobacillales were associated with higher CD4%, less microbial translocation, less systemic immune activation, less gut T lymphocyte proliferation, and higher CD4% in the gut	Restoring the immune function during HIV infection
Kumpu et al. [43]	Nasopharyngeal colonization of respiratory virus in children	—	Shorter duration of symptomatic infection, no difference in the frequency of symptomatic infection/severity of symptoms	—	Not protective from the incidence but effective in shortening duration of symptoms
Verhoeven et al. [44]	HPV related precancerous lesions	No significant difference	—	Twice chance for clearance of cytological abnormalities in pap smear	Enhancement of the clearance of HPV-related cytological abnormalities
Hummelen et al. [45]	Immune function of HIV + patients naive to ART	—	No difference	No difference	Not effective
Nagata et al. [19]	Noroviral gastroenteritis in elderly	—	No difference in the incidence rate, decreased fever duration	—	Not protective from the incidence but effective in shortening the duration of symptoms
Irvine et al. [20]	Mucosal integrity and opportunistic infections of HIV + patients	—	Less GI upset and fever incidence	—	Effective, safe, and tolerable
Grandy et al. [46]	Rotaviral diarrhea in children	—	Decreased duration of diarrhea and fever	—	Safe and effective
Irvine et al. [21]	CD4+ numbers in HIV + patients	—	—	Increased in CD4+ count	Increased CD4+ count
Teran et al. [47]	Rotaviral diarrhea in children	—	Shorter duration of hospitalization and diarrhea in nitazoxanide and probiotics	—	Effective more that ORS per se but less than nitazoxanide
Fang et al. [48]	Rotaviral diarrhea in children	Decreased viral shedding in children receiving high dose LAB	—	—	Dose-dependent efficacy of L. *rhamnosus*
Dubey et al. [49]	Rotaviral diarrhea in children	—	Earlier improved stool consistency and decrease in frequency of defecation, less need to ORS	—	Faster recovery, decreased stool volume losses during diarrhea
Matsuzaki et al. [50]	HTLV-1 associated myelopathy/tropical spastic paraparesis	No significant difference in provirus count	Improved spasticity and urinary symptoms	Increased NK cell activity	Safe and effective
Sarker et al. [51]	Viral diarrhea in children	—	Decreased stool frequency, stool volume, and ORS intake in non rotaviral and less sever rotaviral diarrhea	—	Effective in nonrotaviral diarrhea, but ineffective in rotavirus diarrhea
Salminen et al. [22]	Amelioration of GI symptoms in HIV + patients on HAART	—	No difference	—	Tolerable but without significant effect
Mastretta et al. [52]	Prevention of nosocomial rotavirus infections	—	Not effective	—	Ineffective in the prevention of infection
Wrolf et al. [53]	Safety and tolerability of probiotics in HIV + patients	—	Not significant	Not significant	Safe and tolerable
Majamaa et al. [54]	Acute rotaviral gastroenteritis in children	—	Decreased duration of diarrhea	Increased serum and secretory IgA	Enhancement of the serum and intestinal Ab response against rotavirus

HR-HPV: high risk human papilloma virus, HCV: hepatitis C virus, RSV: respiratory syncytial virus, HSV-2: herpes simplex virus- 2, HIV: human immunodeficiency virus, EBV: Epstein Barr virus, CMV: cytomegalo virus, HRV: human rhinovirus, HEV: human enterovirus, ADV: adenovirus, HTLV-1: human T-lymphotropic virus type 1, Ab: antibody, IL-6: interleukin-6, ESR: erythrocyte sedimentation rate, INF: interferon, NK cell: natural killer cell, ORS: oral replacement solution, IgA: immunoglobin A, ART: anti-retroviral therapy, URTI: upper respiratory tract infection.

## Data Availability

The data used to support the findings of this study are available from the corresponding author upon reasonable request.
